# Influence of recent randomized MeVO trials on current practice patterns and future role of MeVO thrombectomy

**DOI:** 10.1177/15910199251389060

**Published:** 2025-10-25

**Authors:** Salome Lou Bosshart, Yu Zhou, Alexander Stebner, Nima Kashani, Mayank Goyal, Michael Hill, Bijoy Menon, Mohammed Almekhlafi, Aravind Ganesh, Nishita Singh, Andrew Demchuk, Jianmin Liu, Johanna Maria Ospel

**Affiliations:** 1 Department of Neurology, 685817 University Hospital Basel, University of Basel, Basel, Switzerland; 2 Neurovascular Center, 12521 Changhai Hospital, Naval Medical University, Shanghai, China; 3 Department of Radiology, 30262 University Hospital Basel, University of Basel, Basel, Switzerland; 4 Department of Radiology, 2129 University of Manitoba, Winnipeg, Canada; 5 Department of Radiology, 2129 Foothills Medical Centre, University of Calgary, Calgary, Canada; 6 Department of Clinical Neurosciences, 2129 Foothills Medical Centre, University of Calgary, Calgary, Canada; 7 Department of Neurology, 12359 University of Manitoba, Winnipeg, Canada

**Keywords:** MeVO, medium vessel occlusion, endovascular treatment, EVT, mechanical thrombectomy

## Abstract

**Introduction:**

Numerous non-randomized studies suggest the benefit of endovascular thrombectomy (EVT) for medium vessel occlusion (MeVO) stroke, while recent randomized trials showed no benefit. In clinical practice, MeVO management remains heterogeneous. We gauged the current status quo of endovascular MeVO management and physicians’ perspectives on future MeVO-EVT trials.

**Methods:**

International, cross-sectional survey study inquiring about practice patterns before and after publication of the MeVO trials, opinions on the design of second-generation MeVO trials, willingness to enroll in such trials, and personal opinions regarding optimal MeVO treatment strategies. Participants’ anonymized responses were summarized using descriptive statistics.

**Results:**

Four hundred fifty-six physicians responded to the survey (308 China, 55 North America, 48 Europe, and 45 other countries). 86/454 (18.9%) respondents reported treating more MeVOs with EVT since the recent MeVO-EVT trials, and 134/454 (29.5%) reported treating fewer. Four-hundred-sixteen of 454 (91.6%) reported that their willingness to enroll patients in a second-generation MeVO-EVT trial increased (282/454 [62.1%]) or remained the same (134/454 [29.5%]) compared to before the recent trial publications. Of 122/454 (26.9%) respondents who participated in a MeVO-EVT trial, 79/121 (65.3%) stated that enrolment bias occurred at their sites. Three hundred twelve of 454 (68.7%) respondents (271/378 [71.7%] interventionalists, 41/76 [53.9%] non-interventionalists, *p* = 0.004) expressed belief in EVT benefit despite the neutral trial results. Adjunct intra-arterial thrombolysis was anticipated to play a major role in future MeVO treatment by 290/454 (63.9%).

**Conclusion:**

Most physicians think that EVT is beneficial in selected MeVO patients. Enrolment bias was thought to be a major contributor to the neutral trial results. Ninety percent were equally or more willing to enroll patients in a second-generation MeVO-EVT trial. Adjunctive intra-arterial thrombolytics were identified as a key strategy for future MeVO-EVT.

## Introduction

In 2015, several randomized controlled trials provided evidence for the safety and efficacy of endovascular thrombectomy (EVT) in large-vessel occlusion stroke.^1^ Some of these trials included proximal M2 occlusions, but the benefit of EVT for medium vessel occlusions (MeVOs), that is, occlusions of the distal M2 and M3 segments, A2 and A3 segments, and P2 and P3 segments, was uncertain.^2^ Although non-randomized data suggested a possible benefit of EVT in MeVO stroke,^3^ no randomized data were available to support MeVO-EVT, and decisions for or against EVT in stroke patients with acute stroke due to MeVO were mostly made on a case-by-case basis and often depended on the interventionalist's experience, comfort level, and technical skills. In early 2025, 10 years after the pivotal LVO-EVT trials, two major randomized controlled trials investigating the benefit of MeVO-EVT—ESCAPE-MeVO and DISTAL—were published. The ESCAPE-MeVO trial enrolled patients with acute ischemic stroke due to MeVO, defined as occlusions of the nondominant/codominant M2, M3, A2, A3, P2, and P3 segment, within 12 h of last known well and found that EVT did not improve 90-day functional outcome compared to usual care, and was associated with a higher rate of symptomatic intracranial hemorrhage and mortality.^4^ Similarly, the DISTAL trial, which included patients with occlusions of the nondominant/codominant M2, M3, M4, A1–A3, and P1–P3 segments up to 24 h after last known well, found no significant difference in functional status or mortality at 90 days between patients randomized to EVT in addition to usual care versus those randomized to usual care only.^5^ Preliminary data from a third MeVO-EVT trial (DISCOUNT, interim analysis presented at ISC 2025) showed comparable results, and in fact suggested a possibility of harm in the EVT arm due to hemorrhagic complications.

However, some believe that the neutral results of the above trials may be related to selective enrolment of patients and lack of equipoise among the treating clinical teams, such that MeVO patients most likely to benefit from EVT may have been treated outside the trials.^6^ Current American and European guidelines, which were drafted prior to publication of these trials, neither recommend nor discourage EVT for MeVO stroke.^7,8^ The clinical reality is that MeVO management patterns are variable, ranging from highly aggressive strategies that treat nearly all MeVOs with EVT, to more conservative approaches in which EVT is hardly used at all. Rather than streamlining the management of EVT in MeVOs, the publication of the recent MeVO-EVT trials has increased controversy and further fueled the discussion on what constitutes “the best treatment approach.”

We conducted a survey among neurointerventionalists and stroke physicians that aimed to gauge the current status of practice patterns for acute stroke due to MeVO after publication of the recent MeVO-EVT trials, and how these trials have changed MeVO management. We also assessed the willingness of neurointerventionalists and stroke physicians to enroll patients in future trials investigating EVT in MeVO patients.

## Methods

This survey study was prepared in accordance with the “Checklist for Reporting of Survey^9^ Studies” (CROSS) of the “Enhancing the QUAlity and Transparency Of health Research” (EQUATOR) network.^10^

We performed an international, cross-sectional survey study among neurointerventionalists and stroke physicians. The questionnaire was developed by a multidisciplinary group of stroke neurologists and neurointerventionalists with expertise in clinical trial design. Items were drafted based on a review of the existing literature and refined through iterative discussions within the study team. The survey was pilot-tested among 10 stroke physicians to assess clarity, comprehensibility, and content coverage. Minor adjustments were made before wide distribution, supporting the face validity of the instrument. Data were acquired through a web-based, anonymous, invite-only survey using Qualtrics (Qualtrics.com), an online survey platform with servers located in Montreal, Canada. The survey employed a convenience-based snowball sampling strategy, whereby the survey link was distributed over several channels, including Linkedin, WhatsApp, the Canadian Stroke Consortium (via mailing list), the Oriental Cerebrovascular Disease Conference (OCIN) 2025 meeting participants (via a QR code), the ESCAPE-MeVO investigators (via mailing list), and through the authors’ personal academic networks. This broad approach was taken to reach as many physicians as possible, of all career stages, and achieve wide representation of the American, European, and Asian physician communities. However, because the exact number of physicians exposed to the survey invitation could not be reliably determined, a formal response rate could not be calculated. Survey responses were collected in an anonymized form.

The questionnaire contained 21 multiple-choice questions inquiring about demographic information, practice patterns before and after publication of the MeVO trials, opinions on the design of second-generation MeVO trials, willingness to enroll in such trials, and personal opinions regarding optimal MeVO treatment strategies (the Supplemental Material). Questions were available to the participants in English and Chinese. Participation in individual survey items was voluntary. Respondents could omit certain questions if they did not feel qualified to provide an answer.

### Statistical analysis

Participants’ characteristics and responses were reported using descriptive statistics, namely counts and frequencies for categorical data, and means and standard deviations for continuous data, and analyzed using Fisher's exact test, as appropriate to the type and distribution of the data. Given the exploratory and descriptive nature of this survey, and the considerable imbalance in group sizes across regions and specialties, and the possibility of confounding factors that may not have been fully captured, no multivariable analyses were performed, since multivariable modeling would likely have been underpowered and potentially misleading. The findings of this study should therefore be regarded as descriptive and hypothesis-generating. All statistical tests were conducted at a significance level of α=0.05 . The statistical analyses were performed using Stata MP 17.0.

## Results

Response data were collected from March to May 2025. A total of 616 participants initiated the survey, of whom 456 completed the questionnaire fully, though individual item response rates varied slightly. The majority of respondents (246/456 [54.0%]) were interventional neurologists, followed by 77/456 (16.9%) endovascular neurosurgeons and 56/46 (12.3%) interventional neuroradiologists. Seventy-seven of 456 (16.9%) were non-interventional stroke physicians. Most participants were from China (308/456 [67.5%]), followed by Europe (48/456 [10.5%]), and Canada (40/456 [8.8%]). The percentage of interventionalists among all respondents was 96.8% for Chinese respondents, 65.4% for respondents from other Asian countries, 81.3% for European respondents, 60% for the USA, and 15% for Canadian respondents. Detailed demographic characteristics of the respondents are shown in Table 1.

**Table 1.** Demographic characteristics of survey participants.

Demographic characteristics	*N* (%) – Total *N* = 456
Age (years) < 30 30–40 41–50 51–60 61–70 > 70	8/445 (1.8%) 173/445 (38.9%) 199/445 (44.7%) 48/445 (13.0%) 6/445 (1.4%) 1/445 (0.2%)
Specialty Stroke neurology (non-interventional) Interventional neurologist Interventional neuroradiologist Diagnostic neuroradiologist Endovascular neurosurgeon Neurosurgeon (non-endovascular) Other	70/456 (15.4%) 246/456 (54.0%) 56/456 (12.3%) 2/456 (0.4%) 77/456 (16.9%) 2/456 (0.4%) 3/456 (0.7%)
Career stage Resident or med student Fellow Junior staff (< 5 years from board certification) Senior staff (> 5 years from board certification)	11/445 (2.5%) 10/445 (2.3%) 44/445 (9.6%) 380/445 (85.4%)
Region Canada Europe USA China Other Asian countries Africa Central/South America	40/443 (9.0%) 48/443 (10.8%) 15/443 (3.4%) 308/443 (69.5%) 26/443 (5.9%) 1/443 (0.2%) 5/443 (1.1%)

### Participation in recent MeVO-EVT trials

Out of 456 respondents, 122 (26.8%) indicated that their hospital participated in a randomized MeVO-EVT trial. Out of these 122 respondents, 42 (34.7%) stated that they enrolled all or almost all eligible patients. Meanwhile, 61 (50.4%) stated that some enrolment bias occurred, and 18 (14.9%) believed there was “a lot of cherry picking” during patient enrollment.

### Changes in practice patterns

Prior to publication of the MeVO-EVT trials, the majority (308/455 [67.7%]) of respondents estimated the number of patients treated for MeVO (defined as distal M2, M3, A2, A3, P2, and P3 occlusions) in their hospital to be < 50 per year.

After publication of the two MeVO trials in February 2025, 220/454 (48.5%) of respondents changed their practice pattern for MeVO stroke patients, but these changes occurred in different directions: 86/454 (18.9%) started to treat more MeVOs with EVT, and 134/454 (29.5%) treated less MeVOs with EVT after the MeVO trial publications. The remaining 234/454 (51.5%) respondents reported that the number of MeVOs treated at their institution did not change. There were distinct geographic differences in how practice patterns changed, as shown in Table 2, whereby only stroke experts practicing in China started to treat more MeVOs after publication of the two MeVO-EVT trials.

**Table 2.** Changes in practice patterns stratified by the geographical region of practice of the respondents.

Geographical region	MeVOs treated after publication of trials—*n* (%)			Total *N*
	More	Equal	Less	
China	83 (27.0)	168 (54.6)	57 (18.5)	308
Europe	0	25 (52.1)	23 (47.9)	48
Canada	0	8 (20)	32 (80)	40
Other Asian countries	2 (7.8)	18 (69.2)	6 (23.1)	26
USA	0	8 (53.3)	23 (46.7)	15
Central/South America	0	4 (80.0)	1 (20.0)	5
Africa	0	1 (100)	0	1
**Total**	85 (19.2)	232 (52.4)	126 (28.4)	443

MeVOs: medium-vessel occlusions; USA: United States of America.

### Beliefs regarding the validity and generalizability of the trial results

Three hundred twelve of 454 (68.7%) respondents felt that the results of the MeVO trials did not reflect the true effect of MeVO-EVT and believed that the EVT may actually be beneficial for MeVO. On the other hand, 142/456 (31.3%) perceived the results of the MeVO trials to be accurate and did not believe that EVT is beneficial in MeVO stroke. Whether the trial results were believed to represent the true EVT effect or differed significantly between interventionalists and non-interventional physicians. While in both groups, more respondents believed that there may still be a benefit of EVT in MeVO stroke despite the trial results, this opinion was more common among interventionalists (271/378 [71.7]%) compared to their non-interventional colleagues (35/76 [54.0%], *p* = 0.004). There were no differences in responses across respondents’ age groups (*p* = 0.258) or career stages (*p* = 0.178).

### Beliefs regarding the future role of EVT in MeVO management

Two hundred fifty-five of 455 (56.0%) of the respondents expressed belief that future trials will find subgroups of MeVO stroke patients who benefit from EVT. Fifty-eight of 455 (12.8%) thought the vast majority of MeVOs will be treated medically, and 77/455 (16.9%) thought most MeVOs will eventually be treated with EVT. With improved tools and treatment strategies, 372/455 (81.8%) of the respondents thought there would be a role for EVT in MeVO stroke treatment. Only 19/455 (4.2%) thought that there would be no role for EVT in MeVO management, and 64/455 (14.1%) were unsure. The majority of respondents (361/454 [9.5%]) believed that mechanical thrombectomy with aspiration only was the most effective endovascular MeVO-EVT strategy. Intra-arterial thrombolysis, in addition to mechanical EVT in MeVO, was believed to play a major role in future MeVO treatment by 290/454 (63.9%) of the survey participants, while only 114/454 stroke physicians saw room for intra-arterial thrombolysis as a stand-alone therapy. Interestingly, 231/454 (50.9%) believed that EVT in combination with intra-arterial thrombolysis was equally safe as either one of the two treatments as a stand-alone therapy.

### Second-generation MeVO-EVT trials

Two hundred eighty-five of 454 (62.1%) respondents stated that, based on the recent MeVO trials, they were more willing to enroll patients into a second-generation MeVO-EVT trial. Willingness to enroll did not change in 134/454 (29.5%), and was lower in only 38/454 (8.4%) of the respondents. Table 3 shows the willingness to enroll stratified by the geographical region of practice of the respondents. There were distinct geographic differences, whereby the willingness to randomize in second-generation EVT trials generally increased more among Asian participants compared to North American and European participants. That said, even in Europe in North America, more than half of the physicians stated that they would be equally or more willing to randomize patients in second-generation trials. The following three changes were identified by the participants as most important to prove the benefit of EVT in a second-generation MeVO-EVT trial:

1. Better reperfusion rates (ranked as most important by 121/453 [26.7%]).
2. Less procedural hemorrhagic complications (ranked as most important by 121/453 [26.7%]).
3. Less non-procedural complications (ranked as most important by 37/453 [8.2%]).

**Table 3.** Willingness to enroll in a second-generation MeVO trial stratified by the geographical region of practice of the respondents.

Geographical region	Willing to enroll in a future MeVO trial—*n* (%)			Total *N*
	More	Equal	Less	
China	220 (71.4)	74 (24.0)	14 (4.6)	308
Europe	19 (39.6)	21 (43.8)	8 (16.7)	48
Canada	11 (27.5)	17 (42.5)	12 (30.0)	40
Other Asian countries	16 (61.5)	8 (30.8)	2 (7.7)	26
USA	8 (53.3)	7 (46.7)	0	15
Central/South America	3 (60.0)	1 (20.0)	1 (20.0)	5
Africa	0	0	1 (100)	1
**Total**	277 (62.5)	128 (28.9)	38 (8.6)	443

MeVO: medium-vessel occlusion; USA: United States of America.

Restricting the trial to a specific EVT technique, treating only patients with more severe deficits, advanced imaging, faster treatment workflows, and better training of the interventionalists were not considered high-priority changes necessary to prove the benefit of EVT in MeVO stroke.

An improvement in mRS 0–1 (excellent functional outcome) rates of 10% (median; IQR 10–20), and a 15% (median; IQR 10–20) improvement of mRS 0–2 (good functional outcome) were considered meaningful to make EVT in MeVO patients worthwhile.

**Figure 1.**
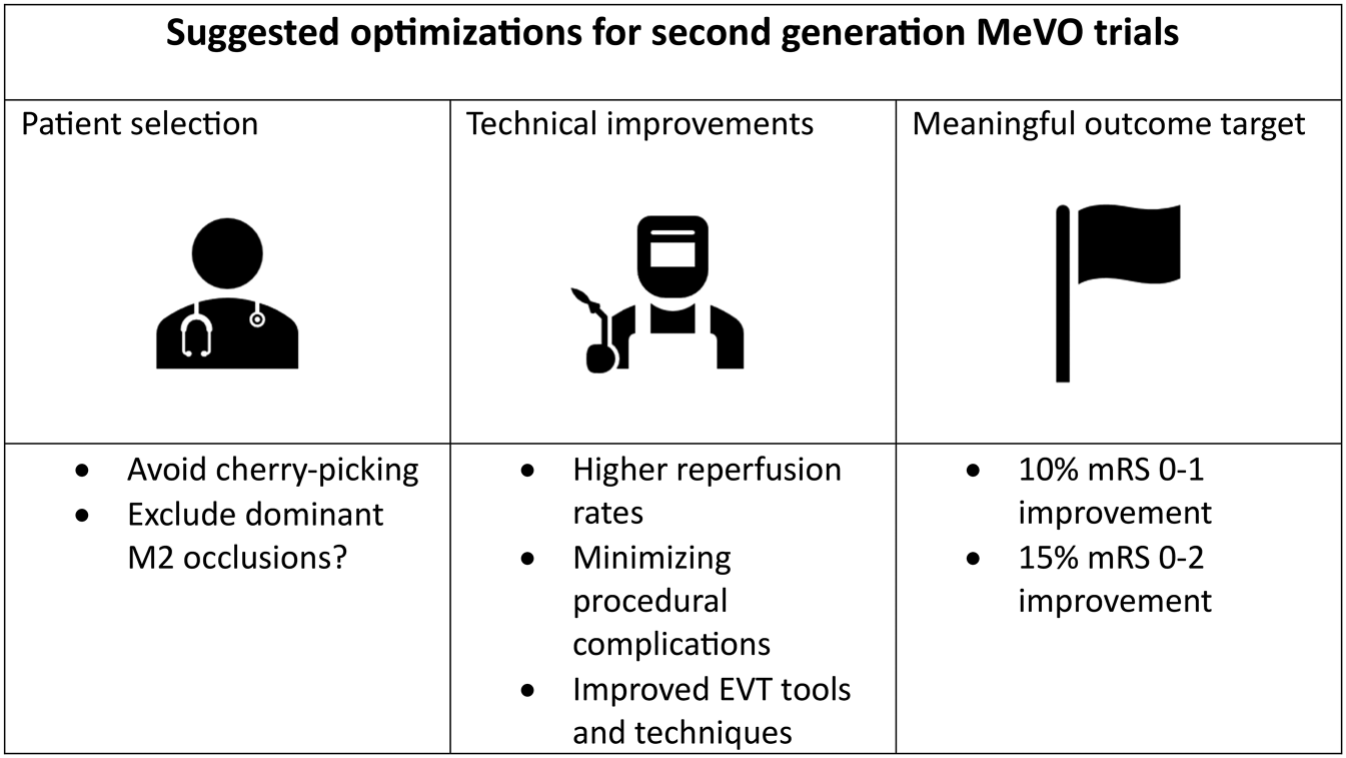
Summary of suggestions by the survey participants for changes in future second-generation medium vessel occlusion (MeVO) trials.

When asked about the definition of MeVO in future trials, 260/454 (57.3%) of the participants thought a dominant M2 occlusion should be considered an LVO and therefore not be randomized in a MeVO trial, while 194/454 (42.7%) thought dominant M2 occlusions should be enrolled. Figure 1 summarizes key opinions of the survey respondents regarding optimal design of next-generation MeVO-EVT trials.

## Discussion

This survey, which collected responses from over 450 stroke physicians—predominantly interventional neurologists—highlights the current uncertainty and variation in the management of acute stroke due to MeVO following the publication of the recent MeVO-EVT trials.

Contrary to prior non-randomized studies that suggested improved outcomes with EVT, the ESCAPE-MeVO and DISTAL randomized trials and DISCOUNT trial interim data did not show the benefit of EVT in patients with acute stroke due to MeVO.^4,5^ In both trials, patient selection was limited to individuals with favorable imaging profiles and clinically significant deficits, thereby potentially excluding those with milder symptoms or less salvageable brain tissue. This restriction, while methodologically appropriate to maximize the likelihood of detecting treatment efficacy in an emerging field, inherently limits the generalizability of the results to the broader MeVO population. Consequently, uncertainty remains for clinicians when considering patients outside these strict inclusion criteria. Naturally, additional research is needed to better delineate which patient subgroups derive meaningful benefit from the intervention. This may be one of the reasons why half of the respondents reported no practice changes in their institutions after the publication of these two trials. Interestingly, among the remaining half, practice patterns shifted in both directions, with some clinicians reporting increased use of EVT and others reporting a reduction. These arbitrary changes in practice patterns may be, at least in part, explained by skepticism towards the trial results, which were expressed by more than two-thirds of the survey participants. Additionally, we observed geographic and specialty-related imbalances in practice patterns. Particularly, the interventionalists believed that EVT may still confer benefit in MeVO stroke. Previous survey studies have shown that neurointerventionalists may be more optimistic regarding interventional stroke treatment than non-interventional stroke physicians.^11^ The reasons for this difference remain speculative; however, it is possible that professional incentives play a role, as a positive trial outcome could expand the scope of practice and increase demand for interventions.

There were also striking geographic patterns with regard to practice changes, whereby stroke experts practicing in China, but not in other geographic regions, reported an increased amount of MeVOs treated after publication of the DISTAL and ESCAPE-MeVO trials and presentation of the DISCOUNT interim data. Because all these trials were conducted in a predominantly Caucasian population, Chinese practitioners may feel that the trial results are not necessarily applicable to their patient population and therefore perhaps do not base their clinical decisions on these data. Interestingly, earlier survey research on the treatment of basilar artery occlusion demonstrated that Chinese interventionalists were more supportive of thrombectomy than their Western colleagues.^11^ In our study, the particularly high proportion of interventionalists among Chinese respondents may therefore explain, at least in part, the strong optimism expressed by this group. These geographic and specialty-related imbalances should be considered when interpreting the generalizability of our findings. Additionally, physicians with strong opinions regarding MeVO-EVT may have been more likely to participate, a potential response bias inherent to survey studies with optional participation.^12^

Conversely, 31.3% of respondents indicated that they accepted the trial results as accurate and did not believe EVT to be beneficial in MeVO stroke. While our survey did not explore the underlying reasons for this view, possible explanations include greater confidence in the methodological rigor of randomized controlled trial evidence compared to non-randomized data, as well as reliance on personal experience that may not have demonstrated consistent benefit. This group also included a higher proportion of non-interventional stroke physicians, who, in our survey, were overall less likely to believe in EVT benefit than interventionalists.

It should be noted that our survey can only establish a temporal, but not a direct causal relationship between the publication of trial results and reported practice changes. Although respondents were asked about practice patterns before and after the trials, other unmeasured factors—such as local or national guideline updates, device availability, reimbursement schemes, institutional policies, or regional practice culture—may also have contributed to these changes.

Most respondents believe that better patient selection and improved interventional tools and techniques are needed to prove the benefit of EVT in MeVO stroke, similar to LVO stroke, where an initial wave of neutral EVT trials was followed by a second series of trials in which improved patient selection criteria and EVT techniques were employed, and EVT benefit could finally be proven.^13^

Observing the optimism of stroke experts towards EVT as a treatment option for MeVO stroke, and their heavy reliance on randomized trial data for treatment decision-making, one may wonder why selective enrolment into MeVO-EVT trials may have been a problem—after all, proving safety and efficacy of MeVO-EVT in a high-quality randomized trial would be the most straightforward way to pave the way for MeVO-EVT becoming standard of care. However, in clinical reality, the decision to enroll a patient in such a trial is not that easy. Almost every stroke physician remembers MeVO stroke patients who drastically improved with EVT and others who remained heavily disabled when no EVT was performed. That being said, there may be some degree of recall bias and we perhaps tend to forget the procedural complications more quickly.

It is indeed well known that physicians have a tendency to choose active treatment rather than a watch-and-wait approach, particularly in situations where there is no clear evidence supporting either option.^14^ This may have led to selective enrloment of patients into the MeVO trials, that is, non-enrollment and “off-label” treatment of patients who were believed to have high chances of benefitting from EVT. Our survey data supports this hypothesis, with two-thirds of the trial participant respondents reporting that at least some degree of enrolment bias occurred at their hospitals-likely compromising the generalizability of the trial results.^15^ While subjective reports do not prove that enrolment bias occurred, they do raise the suspicion that selective enrolment appears to be a principal reason why physicians still are not convinced that EVT should be avoided in MeVO stroke patients. In fact, there seems to be high motivation to generate evidence for EVT as a possible treatment option, with a large proportion of participants stating that their willingness to randomize patients in MeVO-EVT trials has increased after the neutral first-generation MeVO-EVT trial results were published, particularly in China. However, the reports of enrolment bias must be interpreted with caution, as they reflect anecdotal, self-reported perceptions rather than objective evidence of trial conduct. While such responses provide valuable insight into how clinicians interpreted the neutral trial results, they cannot be considered proof that selective enrolment occurred.

Besides failure to consecutively enroll all eligible patients, the survey respondents felt that reperfusion rates were too low to prove the advantage of EVT over best medical treatment in MeVO stroke patients. Indeed, reperfusion success rates (final expanded Thrombolysis in Cerebral Infarction [eTICI] 2b 75% in ESCAPE-MeVO,^4^ 72% in DISTAL^5^) were lower than what is reported for large-vessel occlusion EVT.^1^ Thus, it is no surprise that most survey respondents reported interest in participating in future studies investigating intra-arterial thrombolysis as an adjunct to mechanical thrombectomy to augment reperfusion success rates. The more distal localization and the smaller vessel diameter in MeVO stroke render EVT technically more challenging, and as a result, it is possible that interventionalists may have taken a more cautious approach, refraining from more aggressive maneuvers to improve reperfusion success. In theory, the distal occlusion location could also increase the risk of technical complications, such as vessel rupture, and respondents felt that this risk should ideally be further mitigated in order to improve the efficacy of EVT in MeVO stroke. That being said, technical complication rates in the recent MeVO-EVT trials were low, with vessel perforations occurring in 1%–3%, and symptomatic intracranial hemorrhage rates ranging from 5% to 6% with EVT versus 2% to 3% with usual care.^4,5^ According to the results of this survey, the readiness to enroll patients into second-generation MeVO trials has overall increased since the recent MeVO-EVT trials were published.

## Limitations

This survey study has several limitations. First, more than half of the respondents were neurointerventionalists, which may have skewed responses toward a more neurology-centric perspective. Similarly, since most respondents came from China, the generalizability of the results to other regions may be limited, although there were >100 responses from other geographic regions as well. Second, the survey relies on self-reported changes in practice patterns and perceptions, which are subjective and vulnerable to recall bias and other biases. No quantitative data on MeVO-EVT volumes were collected to objectively validate reported changes in EVT volumes performed in MeVO stroke patients. As responses were collected in a fully anonymized manner, we were unable to verify the accuracy of reported changes at the hospital level. The survey, therefore, reflects physicians’ perceptions of changes in practice patterns rather than documented treatment volumes. Third, since responses were collected in an anonymized fashion, response rates could not be tracked accurately, and the possibility of repeat responses by a single physician could not be excluded with certainty. Fourth, our study relied on descriptive statistics only. Although multivariable analysis could, in theory, provide insight into independent factors associated with practice changes or beliefs, the stark imbalance in group sizes and the possibility of confounding factors (e.g. reimbursement regimens) that were not systematically captured in the survey limited the feasibility and reliability of such an approach. Fifth, the survey distribution approach, while allowing broad international reach, may have introduced selection bias by favoring more engaged physicians or those with stronger opinions about EVT. Sixth, survey items such as those assessing beliefs about EVT were structured as binary or Likert-scale questions, which may not fully capture all nuances of physicians’ reasoning or the spectrum of clinical attitudes. While this format was chosen to facilitate brevity, clarity, and comparability across a large international cohort, it inevitably restricts the depth of responses.

## Conclusion

This international survey showed that more than two-thirds of interventional and non-interventional stroke physicians believe there may be a benefit of EVT in selected MeVO stroke patients, despite the neutral results of the recently published MeVO-EVT trials. Enrolment bias was thought to be a major contributor to the neutral trial results. Nine out of 10 respondents stated they were equally willing or more willing to enroll patients in a second-generation MeVO-EVT trial, particularly in Asia. Adjunctive intra-arterial thrombolysis to improve reperfusion rates was identified as a key strategy to prove EVT benefit in MeVO stroke.

## Supplemental Material

sj-docx-1-ine-10.1177_15910199251389060 - Supplemental material for Influence of recent randomized MeVO trials on current practice patterns and future role of MeVO thrombectomy Supplemental material, sj-docx-1-ine-10.1177_15910199251389060 for Influence of recent randomized MeVO trials on current practice patterns and future role of MeVO thrombectomy by Salome Lou Bosshart, Yu Zhou, Alexander Stebner, Nima Kashani, Mayank Goyal, Michael Hill, Bijoy Menon, Mohammed Almekhlafi, Aravind Ganesh, Nishita Singh, Andrew Demchuk, Jianmin Liu and Johanna Maria Ospel in Interventional Neuroradiology
