# Supplementary material for: Influence of recent randomized MeVO trials on current practice patterns and future role of MeVO thrombectomy
Source: Interv Neuroradiol. 2025 Oct 25:15910199251389060. Online ahead of print. doi: 10.1177/15910199251389060 (PMC12553542; doi:10.1177/15910199251389060)
Supplement: sj-docx-1-ine-10.1177_15910199251389060 - Supplemental material for Influence of recent randomized MeVO trials on current practice patterns and future role of MeVO thrombectomy [file sj-docx-1-ine-10.1177_15910199251389060.docx]

Post-trial MeVO practice patterns

Start of Block: Dear colleagues,

Q1 Dear colleagues and friends, This is a **very short 3-5 minute survey** to gauge your practice patterns for acute ischemic stroke due to Medium Vessel Occlusion (MeVO) in the post-MeVO trial era. We would like to know your current treatment approach to MeVOs and **whether you have changed your approach based on the recently presented MeVO trial results.** In answering this survey, your consent to use your data is implied. We will de-identify all responses. Your implied consent means that your responses will be recorded in an electronic database and will be used for future research, including publications or grant applications. Your participation is much appreciated and will help guide further research to improve outcomes in patients with MeVO stroke! Let's start with your hospital. **Did your hospital participate in one of the MeVO trials?**

- Yes (1)
- No (2)

Q1 **尊敬的主任：** **我们拟开展一项关于中等血管闭塞性卒中（MeVO）的调查，旨在了解MeVO各临床研究发表后，领域内专业人士对MeVO进行血管内治疗（EVT）的态度。** **鉴于您在脑血管病诊疗中的突出贡献，特邀请您参加该问卷填写。您的回答将有助于指导进一步的研究，改善MeVO中风患者的治疗效果，诚挚地感谢您的支持与付出！** **上海长海医院刘建民教授团队**   **您的医院是否参与了MeVO试验？**

- 是 (1)
- 否 (2)

Display this question:

If Dear colleagues and friends,This is a very short 3-5 minute survey to gauge your practice patterns = Yes

Q15 Do you think you **sequentially enrolled all or nearly all eligible MeVOs** in your hospital?

- Yes (1)
- No (there was some degree of "cherry picking") (2)
- No (there was a lot of "cherry picking") (3)

Q15 **您的医院是否按顺序入组了所有或几乎所有符合条件的中MeVO病例？**

- 是 (1)
- 否（存在一定程度的“挑选”） (2)
- 否（存在大量的“挑选”） (3)

Q12 What is your **specialty?**

- Stroke neurology (non-interventional) (1)
- Interventional neurologist (2)
- Interventional neuroradiologist (3)
- Diagnostic neuroradiologist (4)
- Endovascular neurosurgeon (5)
- Neurosurgeon (non-endovascular) (6)
- Other (7)

Q12 **您的专业是什么？**

- 神经内科医生（不从事介入治疗） (1)
- 神经内科医生（从事介入治疗） (2)
- 神经放射学-神经介入 (3)
- 神经放射学-影像诊断 (4)
- 神经外科（从事介入治疗） (5)
- 神经外科（不从事介入治疗） (6)
- 其他 (7)

Q22 What is your age (in years)?

- <30 (1)
- 30-40 (2)
- 40-50 (3)
- 50-60 (4)
- 60-70 (5)
- >70 (6)

Q22 **您的年龄是多少（以年为单位）?**

- 小于30岁 (1)
- 30-40岁 (2)
- 40-50岁 (3)
- 50-60岁 (4)
- 60-70岁 (5)
- 大于70岁 (6)

Q23 What is your career stage?

- Resident or med student/ intern (1)
- Fellow (2)
- Junior staff ( (3)
- Senior staff (>5 years from board certification (4)

Q23 **您的职业阶段是什么？**

- 住院医师或医学生/实习生 (1)
- 专陪人员（Fellow） (2)
- Junior Staff（工作5年内） (3)
- Senior staff（工作超过5年) (4)

| 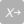 |
| --- |

Q24 What is your country of practice?

▼ Afghanistan (1) ... Zimbabwe (1357)

Q24 **您的执业国家是哪里？**

▼ Afghanistan (1) ... Zimbabwe (1357)

Q3 **How many MeVOs** (defined as distal M2, M3, A2, A3, P2, P3 occlusions) were appproximately **treated per year in your hospital prior to the MeVO EVT trials** (this includes EVT and intravenous thrombolysis)?

- <50 (1)
- 50-100 (2)
- 100-150 (3)
- 150-200 (4)
- >200 (5)

Q3 **在MeVO EVT各临床试验之前，您所在医院每年大约治疗了多少例MeVO（定义为远端M2、M3、A2、A3、P2、P3闭塞）（本治疗包括动脉内治疗和静脉溶栓）？**

- 小于50例 (1)
- 50-100例 (2)
- 100-150例 (3)
- 150-200例 (4)
- 大于200例 (5)

| Page Break |  |
| --- | --- |

Q4 Above are the **key results of the three recently presented MeVO trials** (presented at ISC in February 2025). Are you now treating **more, equal numbers or less MeVOs with EVT** in your clinical practice **because of these results?**

- I treat more MeVOs with EVT because of these results (1)
- The results have not changed my practice and I treat the same number of MeVOs with EVT (2)
- I treat less MeVOs with EVT because of these results (3)

Q4 **以上是三项 MeVO研究的关键结果（发布于2025年2月在国际卒中大会ISC）。** **在这些结果发布后，您现在对 MeVO 患者进行血管内治疗的数量是增多了、保持不变还是减少了呢？**

- 对 MeVO 患者进行EVT的数量比以往更多了 (1)
- 这些结果并没有改变我的做法，对 MeVO 患者进行EVT的数量保持不变 (2)
- 基于这些结果，对 MeVO 患者进行EVT的数量较前减少了 (3)

Q19 Do you personally think that these MeVO EVT trials **reflect reality** and there **truly is no benefit of EVT in MeVO stroke?**

- The results are accurate, there truly is no benefit of EVT in MeVO stroke (1)
- The results are not accurate. There is benefit of EVT in MeVO stroke (2)

Q19 **在您看来，这些MeVO的临床试验是否反映了EVT的真实情况？ MeVO卒中患者进行EVT是否真的没有获益吗？**

- 结果是准确的，MeVO卒中患者进行EVT没有获益 (1)
- 结果不是准确的，MeVO卒中患者进行EVT有获益  (2)

Q13 **Which changes would need to be made** in a second generation MeVO EVT trial to prove benefit of EVT? Rank in **order of importance.**

______ Better reperfusion rates (meTICI 2b 75% in ESCAPE-MeVO, 72% in DISTAL) (1)

______ Less procedural hemorrhages (sICH EVT vs. control arm 5% vs. 2% in ESCAPE-MeVO, 6% vs. 3% in DISTAL) (2)

______ Less non-procedural complications (eg, pneumonia, other/ anesthesia-related complications. Total SAE 34% vs. 26% in ESCAPE-MeVO, 42% vs. 32% in DISTAL) (3)

______ Faster treatment workflow times (treat only patients presenting in the early time window. Median onset-to-arterial puncture time 5.2 h in ESCAPE-MeVO, 4.9 h in DISTAL) (4)

______ Better training/ more experience of operators (no strict training/ minimum volume requirements in any of the MeVO trials) (5)

______ Only treat MeVOs with more severe deficits (high NIHSS: enrolment of NIHSS >5 or even 3-5 with disabling deficits in ESCAPE-MeVO, >3 in DISTAL) (6)

______ Ensure that there is salvageable tissue with advanced imaging (eg, CTP, multiphase CTA, MRI: advanced imaging not mandated in any of the MeVO trials) (7)

______ Restricting the trial to a specific EVT technique (stent-retriever EVT mandatory in ESCAPE-MeVO, aspiration only or stent-retriever EVT used in DISTAL and DISCOUNT) (8)

Q13 **如果再进行MeVO的EVT研究，您觉得应该在哪些方面进行改进才可能使患者在EVT治疗中获益？请您按重要性排序。**

______ 更高的血管再通率（在ESCAPE-MeVO试验中eTICI 2b级为75%，在DISTAL试验中为72%） (1)

______ 减少手术相关出血（在ESCAPE-MeVO试验中，EVT组症状性颅内出血发生率为5%，对照组为2%；在DISTAL试验中分别为6%和3% ） (2)

______ 减少非手术相关并发症（如肺炎、其他/麻醉相关的并发症。在ESCAPE-MeVO试验中，严重不良事件总发生率为34%，对照组为26% ；在DISTAL试验中分别为42%和32% ） (3)

______ 加快治疗流程时间（比如仅治疗在早期时间窗内就诊的患者。在ESCAPE-MeVO试验中，发病至动脉穿刺的中位时间为5.2小时，在DISTAL试验中为4.9小时） (4)

______ 操作人员接受更好的培训/积累更多经验（在之前MeVO试验对操作人员没有严格的培训要求或最低手术量要求） (5)

______ 仅治疗神经功能缺损更严重的MeVO患者（比如纳入高NIHSS评分的患者：在ESCAPE-MeVO试验中，纳入的NIHSS评分>5分，甚至对于存在致残性缺损的患者评分≥3-5分；在DISTAL试验中，NIHSS评分>3分 ） (6)

______ 通过高级影像学（如CT灌注成像[CTP]、多时相CT血管造影（CTA）、磁共振成像[MRI]）确认存在可挽救的脑组织（目前在任何MeVO试验中都没有强制要求使用高级影像学检查） (7)

______ 将试验限制在特定的EVT技术（在ESCAPE-MeVO试验中，必须使用支架取栓术；在DISTAL和DISCOUNT试验中，可以使用单纯抽吸或支架取栓术） (8)

Q5 Based on the recently published/presented MeVO EVT trial results, would you be more willing, less willing or equally willing to **randomize MeVO patients into such a second-generation MeVO EVT trial** (eg, a trial with improved tools, a different time window and modified inclusion criteria?)

- More willing to randomize into a MeVO EVT trial compared to before (1)
- Equally willing to randomize into a MeVO EVT trial compared to before (2)
- Less willing to randomize into a MeVO EVT trial compared to before (3)

Q5 **如果后续进一步开展改良后的MeVO临床研究（例如使用更佳的取栓工具、不同时间窗和修改后的纳入标准），您对参加临床实验、对患者进行随机的意愿如何？**

- 与之前相比，更愿意将患者随机纳入MeVO EVT临床研究 (1)
- 与之前相比，愿意程度相同 (2)
- 与之前相比，更不愿意将患者随机纳入MeVO EVT临床研究 (3)

Q16 Do you think **proximal, dominant M2 occlusions** should be considered MeVOs and get randomized in such a trial?

- Yes (1)
- No, they should be considered LVOs and NOT get randomized (2)

Q16 **在您看来，大脑中动脉M2主干、近端闭塞是否应被视为MeVO，并纳入此类试验进行随机分组？**

- 是，应该被认为是MeVO，可以入组 (1)
- 不，它们应被视为大血管闭塞（LVO），不能入组  (2)

| Page Break |  |
| --- | --- |

Q14 For MeVO EVT, what improvement in **90-day mRS 0-1 rates would be meaningful in your opinion to make EVT worthwhile?** *For an improvement of 20% or higher, select slider position at 20%.*

|  | 0 | 1 | 2 | 3 | 4 | 5 | 6 | 7 | 8 | 9 | 10 | 11 | 12 | 13 | 14 | 15 | 16 | 17 | 18 | 19 | 20 |
| --- | --- | --- | --- | --- | --- | --- | --- | --- | --- | --- | --- | --- | --- | --- | --- | --- | --- | --- | --- | --- | --- |

| Percent mRS improvement () | 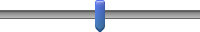 |
| --- | --- |

Q14 **在您看来， 90天mRS 0-1分比例应该提高多少（与非EVT相比），才值得对MeVO进行EVT治疗？** 如果提高幅度为20%或更高，请将滑块移至20%的位置。

|  | 0 | 1 | 2 | 3 | 4 | 5 | 6 | 7 | 8 | 9 | 10 | 11 | 12 | 13 | 14 | 15 | 16 | 17 | 18 | 19 | 20 |
| --- | --- | --- | --- | --- | --- | --- | --- | --- | --- | --- | --- | --- | --- | --- | --- | --- | --- | --- | --- | --- | --- |

| Percent mRS improvement () | 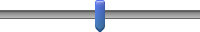 |
| --- | --- |

Q20 For MeVO EVT, what improvement in **90-day mRS 0-2 rates would be meaningful in your opinion to make EVT worthwhile?** *For an improvement of 20% or higher, select slider position at 20%.*

|  | 0 | 1 | 2 | 3 | 4 | 5 | 6 | 7 | 8 | 9 | 10 | 11 | 12 | 13 | 14 | 15 | 16 | 17 | 18 | 19 | 20 |
| --- | --- | --- | --- | --- | --- | --- | --- | --- | --- | --- | --- | --- | --- | --- | --- | --- | --- | --- | --- | --- | --- |

| Percent mRS improvement () | 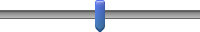 |
| --- | --- |

Q20 **90天mRS 0-2分比例应该提高多少（与非EVT相比），才值得对MeVO进行EVT治疗？** 如果提高幅度为20%或更高，请将滑块移至20%的位置。

|  | 0 | 1 | 2 | 3 | 4 | 5 | 6 | 7 | 8 | 9 | 10 | 11 | 12 | 13 | 14 | 15 | 16 | 17 | 18 | 19 | 20 |
| --- | --- | --- | --- | --- | --- | --- | --- | --- | --- | --- | --- | --- | --- | --- | --- | --- | --- | --- | --- | --- | --- |

| Percent mRS improvement () | 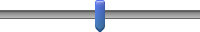 |
| --- | --- |

| Page Break |  |
| --- | --- |

Q6 Where do you think the **future of MeVO treatment** is?

- The vast majority of MeVOs will be treated medically (IV thrombolysis if eligible, otherwise supportive treatment) (1)
- Some MeVOs will be treated with EVT, others will be treated medically (2)
- Most MeVOs will eventually be treated with EVT (3)
- I am not sure. We need additional trials to find this out. (4)

Q6 **您认为MeVO治疗的将来会是什么样子的？**

- 绝大多数MeVO患者都将接受药物治疗（符合条件的进行静脉溶栓治疗，否则进行支持性治疗） (1)
- 部分MeVO患者将接受EVT治疗，其余患者接受药物治疗 (2)
- 大多数MeVO患者将最终接受EVT治疗 (3)
- 不确定未来是怎么样的，我们需要更多的临床试验 (4)

Q7 Do you think that, with improved tools and treatment strategies, there is a **role for EVT in MeVO stroke?**

- Definitely not (1)
- Probably not (2)
- Might or might not (3)
- Probably yes (4)
- Definitely yes (5)

Q7 **如果对血管内治疗的策略和器具进行改进，EVT是否可能在MeVO的治疗有一席之地呢？**

- 肯定不可能 (1)
- 大概不可能 (2)
- 或许能，或许不可能 (3)
- 大概能 (4)
- 肯定能 (5)

Q8 Do you think that there is a **role for intra-arterial thrombolysis in addition to mechanical EVT** in MeVO stroke?

- Definitely not (1)
- Probably not (2)
- Might or might not (3)
- Probably yes (4)
- Definitely yes (5)

Q8 **除了机械性的取栓治疗外，动脉内溶栓治疗在MeVO的治疗中是否也能有一席之地呢？**

- 肯定不可能 (1)
- 大概不可能 (2)
- 或许能，或许不可能 (3)
- 大概能 (4)
- 肯定能 (5)

Q9 Do you think that there is a role for **intra-arterial thrombolysis as a stand-alone treatment** in MeVO stroke?

- Definitely not (1)
- Probably not (2)
- Might or might not (3)
- Probably yes (4)
- Definitely yes (5)

Q9 **动脉内溶栓治疗是否能作为MeVO的单一治疗手段呢（不进行机械方式取栓）？**

- 肯定不可能 (1)
- 大概不可能 (2)
- 或许能，或许不可能 (3)
- 大概能 (4)
- 肯定能 (5)

Q21 Would you be **willing to randomize MeVO patients** into a trial where **intra-arterial thrombolysis alone** is a **treatment option in one arm?**

- Yes (1)
- No (2)

Q21 **如果某项MeVO临床试验中，有一组治疗方案仅为动脉内溶栓治疗，您是否愿意将患者进行入组和随机呢？**

- 愿意  (1)
- 不愿意  (2)

Q10 What do you think is the **most effective** endovascular MeVO EVT strategy?

- Mechanical thrombectomy with stent retriever only (1)
- Mechanical thrombectomy with aspiration only (4)
- Mechanical thrombectomy (stent-retriever or aspiration or combined approach) supplemented with intra-arterial lytic administration (2)
- Intra-arterial lytic administration only (3)

Q10 **您认为对于MeVO的EVT治疗，最有效的血管再通策略是什么？**

- 只使用支架取栓的机械取栓术 (1)
- 只使用抽吸的机械取栓术 (4)
- 机械取栓术（支架取栓、抽吸或联合方法）辅以动脉内溶栓 (2)
- 只进行动脉内溶栓 (3)

Q11 What do you think is the **safest** endovascular MeVO EVT strategy?

- Mechanical thrombectomy only (1)
- Mechanical thrombectomy supplemented with intra-arterial lytic administration (2)
- Intra-arterial lytic administration only (3)

Q11 **您认为针对大脑中动脉远端闭塞（MeVO）的血管内血栓切除术（EVT），最安全的治疗策略是什么？**

- 只进行机械取栓术 (1)
- 机械取栓术辅以动脉内溶栓 (2)
- 只进行动脉内溶栓 (3)

End of Block: Dear colleagues,
